# P02.25. Investigate physiologic function of meridians by changes in Ryodoraku values with acupuncture, moxibustion, and ice stimulation on Zusanli acupoints

**DOI:** 10.1186/1472-6882-12-S1-P81

**Published:** 2012-06-12

**Authors:** Y Lee

**Affiliations:** 1 China Medical University, Taichung, Taiwan

## Purpose

Pathways and characteristics of meridians have been demonstrated by many studies, but the physiological function is still unclear. The Ryodoraku instrument is used to measure skin electrical resistance, which reflects the bioenergy of meridians. We try to investigate the function of meridians by the change in Ryodoraku values with acupuncture, moxibustion, and ice stimulation.

## Methods

M.E.A.D. was used as a measurement tool. Thirty healthy volunteers from the University were recruited. We measured once every week and twice every time in a 30 minute interval. In the first week, 30 subjects sat and rested during the interval, which served as the control group. We compared these two measurements in the first week by reliability analysis. In the second week, we stimulated both Zusanli acupoints for 20 minutes with acupuncture during the interval (acupuncture group). In the third week, we stimulated them by ice (ice group). In the fourth week, we stimulated them by moxibustion (moxibustion group). We analyzed the change of Ryodoraku values between the four groups.

## Results

The correlation coefficients are between 0.79854 and 0.93207 in reliability analysis. Ninety-six percent have good to excellent test-retest reliability in the interval of 30 minutes. The average changes of the control group dropped most significantly. The changes in the acupuncture group both rose and dropped. The changes in the ice group dropped in all meridians, especially stomach meridians. The changes in the moxibustion group raised in all meridians, especially urinary bladder meridians.

## Conclusion

To use the Ryodoraku instrument objectively, the operator should pass test-retest reliability to confirm the consistency of the data. The time to measure must be fixed, but it may not be fixed to see the change of Ryodoraku values at the interval of 30 minutes. We might presume that meridians play an important role in body temperature regulation and it is worth further study.

